# Decoding intentions from movement kinematics

**DOI:** 10.1038/srep37036

**Published:** 2016-11-15

**Authors:** Andrea Cavallo, Atesh Koul, Caterina Ansuini, Francesca Capozzi, Cristina Becchio

**Affiliations:** 1 Department of Psychology, University of Torino, Torino, Italy; 2 C’MON, Cognition, Motion and Neuroscience Unit, Fondazione Istituto Italiano di Tecnologia, Genova, Italy; 3 Department of Psychology, McGill University, Montréal, Canada

## Abstract

How do we understand the intentions of other people? There has been a longstanding controversy over whether it is possible to understand others’ intentions by simply observing their movements. Here, we show that indeed movement kinematics can form the basis for intention detection. By combining kinematics and psychophysical methods with classification and regression tree (CART) modeling, we found that observers utilized a subset of discriminant kinematic features over the total kinematic pattern in order to detect intention from observation of simple motor acts. Intention discriminability covaried with movement kinematics on a trial-by-trial basis, and was directly related to the expression of discriminative features in the observed movements. These findings demonstrate a definable and measurable relationship between the specific features of observed movements and the ability to discriminate intention, providing quantitative evidence of the significance of movement kinematics for anticipating others’ intentional actions.

How do we understand the intentions of other people? Is it possible to understand the intentions of others by simply observing their movements? A widely held, but untested assumption is that the motion that people produce is in most, if not in all, cases consistent with a multitude of different intentions^1^,^2^,^3^,^4^. Based on this assumption, an “intractable non-specificity” problem arises from the observer’s perspective^5^. ‘‘If you see someone in the street raise their hand, they could be hailing a taxi or swatting a wasp^6^’’. Or to repeat a more dramatic example by^1^, is the man in the white coat holding a scalpel to a human’s chest intending to perform a grisly murder or a life-saving operation – is he Dr. Jekyll or Mr. Hyde? The supposed multiplicity of mappings between observed movements and intentions should not allow for this differentiation. In other words, in the absence of context-specifying information, an observer should not be able to identify Dr. Jekyll versus Mr. Hyde^7^.

Previous studies that have sought to examine these observations in a laboratory setting have yielded conflicting results. Some studies indicate that, contrary to the non-specificity hypothesis, observers can use early differences in movement kinematics to discriminate intentions^8^,^9^. For instance, Manera *et al*.^8^ showed that in a binary choice design, observers were able to judge whether the agent’s intent in grasping the object was to cooperate with a partner or compete against an opponent. Other investigators, however, had difficulties replicating these initial findings. Naish *et al*.^10^, for example, report that observers were not able to anticipate the intention to eat, or to place an object from the pre-grasp kinematics. It therefore remains controversial whether and to what extent people are able to detect intention from subtle differences in movement kinematics. Moreover, all of these studies relied almost exclusively on indirect evidence of intention-related information, without quantifying this information in the observed movements. Therefore, put simply, the very availability of kinematic information to classify intention remains unclear.

As a way out of this impasse, in the present study, we sought to determine quantitatively the *transparency* of others’ intentions by decomposing the problem of intention detection into two steps. First, to obtain a measure of the specificity of intention information conveyed by movement patterns, we recorded movement kinematics during execution of grasping movements performed with different intents, and used discriminant analysis as a means to quantify available intention information. Having demonstrated that movement kinematics convey enough information to read-out intentions, we next sought to determine the perceptual efficiency of this information, i.e., its usefulness for perception. To this end, using videos of the same movements (see Supplementary Video S1), we performed a comprehensive set of manipulations, combining rigorous psychophysical techniques and classification and regression tree (CART) modeling. This resulted in a new methodology that allowed us not only to measure, but also to systematically manipulate intention transparency.

Our results provide direct and conclusive evidence that observers exploit intention-related information available in movement kinematics. Moreover, they demonstrate for the first time that specific features of the observed movement predict one’s ability to read intention from movement kinematics.

## Results

### Is intention information available in movement kinematics

To measure the intention information available in movement kinematics, we first obtained a set of natural grasping movements by filming 17 naïve participants reaching towards and grasping a bottle with the intent either to pour some water into a small glass (grasp-to-pour) or to drink water from the bottle (grasp-to-drink). To record movement kinematics, we used a near-infrared camera motion capture system. Kinematics parameters of interest (N = 16, see Materials and Methods section) were computed throughout the reach-to-grasp phase of the movement (based on reach onset and grasp offset) at intervals of 10% of the normalized movement time.

A multivariate analysis of variance (MANOVA) performed on the 16 kinematic features with within-subject factors Intention (to-pour vs. to-drink) and Time (from 10% to 100% of movement duration, in 10 steps) revealed a significant main effect of Intention [F_16,144_ = 11.495; *p *< 0.001, partial η^2^ = 0.995] and a main effect of Time [F_16,144_ = 4.106; *p* < 0.001, partial η^2^ = 0.324]. The interaction Intention by Time was also significant [F_16,144_ = 2.210; *p* < 0.001, partial η^2^ = 0.205]. To determine the extent to which grasping movements differed as a function of intention, we then performed a linear discriminant analysis (LDA). The resulting discriminant function accounted for 100% of variance (Function 1 = 100% of variance, eigenvalue: 5.279, canonical correlation: 0.917) and significantly differentiated grasp-to-drink and grasp-to-pour movements (p < 0.001). Leave-one-out cross-validation confirmed that 92.3% of movements were correctly classified into the corresponding intention.

These findings indicate that movement kinematics contain sufficient information to discriminate between intentions. However, the demonstration of *available information*, per se, is not sufficient to establish its *perceptual efficiency*, i.e., the usefulness of this information for perception^11^. Indeed, despite being encoded in movement kinematics, intention information might nevertheless be invisible to observers. Our next question was, therefore, whether observers would be able to classify intention by simply observing grasping movements.

### Provided that the intention information is available in movement kinematics, can observers classify intention from the observation of grasping movements?

To probe observers’ basic capacity to detect intention, we first divided the dataset of grasping movements (512 movements) into two equal subsets (Subset 1 and Subset 2) and, from Subset 1, selected the 100 movements (grasp-to-pour, N = 50; grasp-to-drink, N = 50) that minimized the within-intention distance, i.e., the distance from the mean variate score of each intention. This procedure allowed us to identify, for each intention, 50 representative movements to be used as stimuli for Experiment 1 (see Supplementary Videos S2–S7).

In this experiment, 18 new participants watched videos of grasping movements. All videos were occluded at the time of contact of the fingers with the bottle. After each video, participants were asked to indicate, in a one-interval discrimination design^12^, whether the observed movement was performed with the intention to drink or to pour from the bottle. After each response, they were then asked to rate how confident they felt in their decision on a 4-point scale (from 1 = least confident, to 4 = most confident).

**Figure 1:**
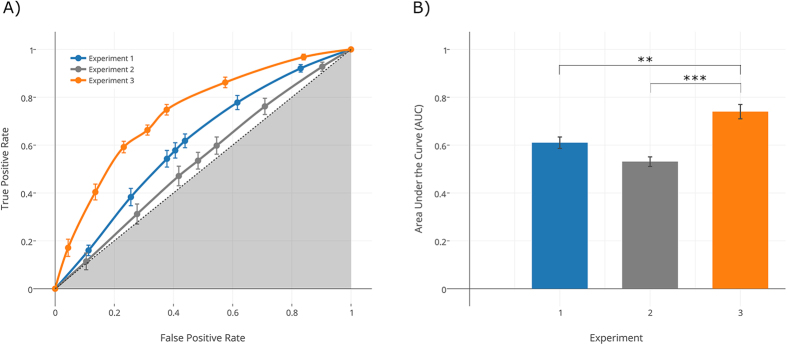
Classification of intention from movement kinematics. Panel (A) The 7-points ROC curves derived from participants’ ratings show probability of true positive rate (hit) versus false-positive rate (false alarm) for grasp-to-pour and grasp-to-drink movements in Experiment 1 (blue), Experiment 2 (gray), and Experiment 3 (orange). The discrimination ability increases as the ROC curve moves from the diagonal (dashed line corresponding to 0.5 random guess performance) towards the upper left boundary of the graph (1.0 perfect performance). Panel (B) Statistical comparisons between AUC values for Experiment 1, 2, and 3. AUC was significantly greater for participants exposed to movements for which the model predicted a higher classification accuracy (Experiment 3) compared to both Experiment 1 (p = 0.001) and Experiment 2 (p < 0.001). No difference emerged from the comparison between Experiments 1 and 2 (p = 0.105). Bonferroni correction was applied to post hoc comparisons.

We used participants’ responses and confidence ratings to estimate Signal Detection Theory parameters. The proportion of hits and false alarms was calculated for each participant, and combined with confidence ratings to determine points on an empirical receiver operating characteristic (ROC) curve. Because each response had four associated ratings, there were eight possible responses for each trial (from highest confidence in one alternative to highest confidence in the other), resulting in seven points on the ROC curve^13^. To obtain an unbiased measure of decoding accuracy, we plotted the points on the ROC curve to determine the area under the curve (AUC) for each participant. AUC values thus generated were significantly above the 0.5 chance threshold (t_17 _= 4.486; p < 0.001; Mean ± SE = 0.608 ± 0.024; 95% C.I. = 0.595–0.621; Fig. 1). This analysis indicates that participants were able to classify intentions from the observation of occluded grasping movements just using the available kinematic information.

### What features do observers use to detect intention?

We proceeded to investigate which specific features participants used for intention classification. To this aim, we trained and tested a Classification and Regression Tree (CART) model, using the 16 kinematic parameters of interested as predictors (see ‘‘Motion capture and video recording’’ section) and the intention choice by participants (to pour vs. to drink; 7114 responses; 1.2% missed responses) as a categorical outcome. We trained the model using 4982 trials (70% of the data), and tested it on the remaining 2132 trials (30% of the data). The generated CART model is depicted in Supplementary Figure S1.

**Figure 2:**
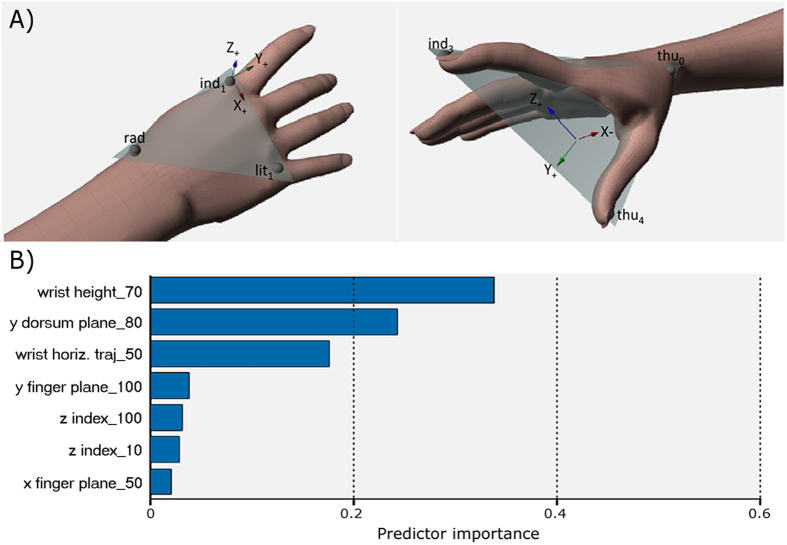
Kinematic determinants of intention choice. Panel (A) (obtained from ^30^*) represents the frontal and lateral view of the marker positions used to characterize kinematics of grasp-to-pour and grasp-to-drink actions. Additional markers (not used to compute the variables of interest) were placed on the metacarpal and proximal interphalangeal joints of the thumb, the proximal interphalangeal joint of the index finger, and the proximal interphalangeal joint and the tip of the little finger. Panel (B) shows the relative importance of kinematic parameters (with respect to normalized movement duration) in determining the intention choice. The wrist height is defined as the z-component of the rad marker; the dorsum plane is defined on the local frame of reference (F_local_) determined by using the markers rad, ind_1_, and lit_1_; the wrist horizontal trajectory corresponds to the x (left-right) component of the rad marker; the finger plane is defined as x, y, and z components of the thumb – index plane that passes through the markers thu_0_, ind_3_, and thu_4_; z-index is defined as the z-coordinate of ind_3_ marker with respect to F_local_. *The image of Panel A was obtained from an open access article distributed under the terms of the Creative Commons Attribution (CC BY) License (https://creativecommons.org/licenses/by/4.0/). CC BY License permits unrestricted use, distribution, and reproduction in any medium, provided the original author and source are credited. Compared to the original image, no changes were made.

The accuracy of the model in predicting participant response was 60.33% for the testing dataset. The corresponding sensitivity and specificity of the model were 65.57% and 44.74%, respectively. The relative importance of kinematic parameters in determining intention choice is depicted in Fig. 2. Wrist height at 70% of movement duration, together with the y-component of the dorsum plane at 80% of movement duration, and the x-component of the wrist trajectory at 50% of movement duration emerged as the most important determinants of intention choice. As depicted in Supplementary Figure S1, the probability that the observed movement was classified as grasp-to-pour was indeed highest when wrist height was lower than 142.56 mm from the table surface, and the y-dorsum plane was lower than −0.284. On the other hand, the probability that the grasping was classified as ‘‘to drink’’ was highest when the wrist height was higher than 142.56 mm, and the x-component of the wrist trajectory deviated by more than 658.35 mm from the left edge of the table (i.e. the volume origin).

### Does the CART generalize to the entire distribution of movements?

Determinants of intention choice in Experiment 1 were estimated on a specific subset of representative motor solutions, i.e., those movements that minimized the within-intention distance. For any motor task, however, there are generally a large number of motor-equivalent solutions that can produce functionally equivalent behaviors^14^. In Experiment 2, we therefore tested the robustness of our results by examining whether the same determinants would generalize across movement patterns. We exposed 17 new participants to the entire distribution of movements in Subset 1 (grasp-to-pour, N = 128; grasp-to-drink, N = 128), including the 100 representative movements used in Experiment 1. Although AUC values for these 100 movements closely replicated those of Experiment 1 (t_33_ = 1.408, p = 0.169), when considering the entire Subset 1 distribution, decoding scores did not exceed the chance level (t_16_ = 1.546, p = 0.142; AUC mean ± SE = 0.531 ± 0.020; 95% C.I. = 0.489–0.573; Fig. 1). To examine whether determinants of intention choice estimated in Experiment 1 were nonetheless able to predict participants’ responses, we then used the generated CART model to predict intention choice over the distribution and compared the actual classification accuracy by participants with the accuracy of classification predicted by the model.

Notably, we found no difference between participants’ actual performance (% of correct responses mean ± SE = 0.519 ± 0.015; 95% C.I. = 0.487–0.551) and the performance predicted by the model (0.549; t_16_ = −2.014; p = 0.061). Additionally, to refine our investigation of the predictive performance of the model, we tested the within participant correlation between the mean accuracy of classification for each of the 256 videos versus the accuracy values predicted by the CART. We expected a positive correlation between these two quantities to the extent that estimated determinants of intention choice reflect knowledge about the natural statistics of human action. Indeed, we observed a strong positive correlation (r_15_ = 0.572; p < 0.01), indicating a successful trial-by-trial basis prediction capability of the CART model. We therefore concluded that, although estimated on a specific subset of motor solutions, the determinants of intention choice can be generalized over the entire distribution of movements predicting both correct and incorrect choices.

### Is it possible to manipulate intention visibility?

Having established a measurable relationship between the kinematics of the movements being viewed and the readability of intentions in those movements, we asked whether it would be possible to improve the ability to detect intention by modulating the expression of kinematic features. We reasoned that if observers use a subset of discriminant features to classify intention, then presenting participants with movements that express these features should aid intention classification. With this in mind, in Experiment 3, we used the CART model to select the 100 grasping movements (grasp-to-pour, N = 50; grasp-to-drink, N = 50) from previously unused Subset 2 for which the CART model predicted the highest classification accuracy, and repeated our measurements on 17 new participants exposed to the corresponding videos.

A t-test showed no difference between the classification accuracy predicted by the CART model (0.697) and that of the participants (mean ± SE = 0.677 ± 0.024, 95% C.I. = 0.626–0.729; t_16 _= −0.814; p =0.427). To further substantiate this finding, we plotted SDT parameters on an ROC curve, and compared corresponding AUC values with those obtained in Experiment 1 and Experiment 2. Univariate ANOVA showed a main effect of Experiment (F_2,49_ = 17.812, p < 0.001, partial η^2^ = 0.421), resulting from significantly greater AUC values for participants exposed to movements for which the model predicted a higher classification accuracy (Experiment 3) compared to AUC values obtained in Experiment 1 (p = 0.001) and Experiment 2 (p < 0.001; Fig. 1B). No difference was found between Experiments 1 and 2 (p = 0.105). This indicates that intention visibility can indeed be directly manipulated by modifying the kinematic parameters of the movements being viewed.

## Discussion

There has been a longstanding controversy as to whether it is possible to detect the intentions of others by simply observing their movements. Our quantitative behavioral experiments demonstrate for the first time that specific features of the observed movement predict the ability to read intention from movement kinematics. In support of this conclusion, we provide evidence from an exhaustive program of experiments designed not only to quantify intention-related information in movement kinematics, but also to measure and manipulate the perceptual efficiency of this information, i.e., its usefulness for the perceptual detection of intention. Remarkably, we found that observers made use of a subset of discriminant kinematics features over the total kinematic pattern in order to discriminate intentions from the observation of simple motor acts. Intention discriminability covaried with movement kinematics on a trial-by-trial basis, and was directly related to the expression of discriminant features in the observed movements. When we examined the possibility to manipulate intention visibility, we found that perceptual detection of intention was indeed higher for movements that expressed these discriminant features. These findings clearly demonstrate a definable and measurable relationship between the specific features of observed movements, as well as the ability to discriminate intention. This further provides direct quantitative evidence of the significance of movement kinematics for anticipating others’ intentional actions.

No current model of intention decoding incorporates such a sophisticated degree of advance information pickup from observed movement patterns. Based on the motor organization of the inferior parietal lobule (IPL)^15^, it has been proposed that, during action execution, IPL neurons coding a given motor act are functionally tuned for a given goal, and therefore linked with specific sets of neurons coding the subsequent motor act. Thus, for example, grasp-to-eat neurons discharge vigorously when grasping is followed by bringing a piece of food to the mouth, and less so when grasping is followed by placing the piece of food into a box. This action chain mechanism has been proposed to facilitate the selection of an impending motor act during action execution^15^. Since the great majority of goal-constrained neurons show the same specificity during execution and observation, the same mechanism would also support the decoding of others’ actions, linking an observed motor act (e.g., grasp) to the motor chain to which it belongs (e.g., grasp-to-eat)^15^,^16^,^17^,^18^,^19^. Thus, this chained representation of an action would endow the observer with the ability to anticipate the agent’s intention in performing a specific motor act (e.g., eating).

A question remains: how can action observation activate the appropriate chain? After all, the observer only ever sees a hand grasping an object^15^. For the action chain model to predict intention, it is therefore necessary to postulate a ‘selection mechanism’ that chooses the correct neuronal chain^18^.

It has been proposed that one way by which this mechanism might operate is based on the context and the object type of the action^15^,^18^. The presence of an empty glass close to the bottle, for example, may signal the agent’s intention to pour from the bottle. Similarly, if the agent has already filled other empty glasses on the table, observers may anticipate the agent’s intention to fill the remaining empty glasses. The match between intention, object, and context, however, is obviously not mandatory. First, intentions are not always triggered in straightforward ways by the environment. As noted by^20^, to suppose otherwise is to ignore the very phenomena, planning, and decisions, which make intention understanding so interesting. Second, within the same context, the same stimulus is often compatible with more than one intention, making the selection of neural chain a complex, underdetermined, inverse problem.

By demonstrating that discriminant kinematic features of the observed grasping can predict the subsequent motor act, our findings strengthen the notion of motor chaining, and suggest that subtle differences in the observed movement kinematics may be useful in determining the most probable chain. Of note is that, on this account, the mechanism supporting chain selection would be *internal*, rather than *external* to the observed motor act. In other words, the kinematic features of the observed act would lead to the activation of the most appropriate neuronal chain. If this is correct, then even in absence of contextual information, IPL mirror neurons should respond differently to the observation of the same motor act, depending on the final goal of the action.

Of course, this does not exclude a role for context. A reasonable framework is that, prior to movement onset, a range of possible intentions is estimated from the spatial and temporal context, arguably in areas outside the mirror system^4^. This prior prediction would impact the process of chain selection, constraining the number of possible intentions^21^. Early on in the movement, discriminant kinematic features of the observed motor act would then lead to the selection of the most probable intention^22^.

This conceptual framework might help explain why individuals with autism have difficulties in comprehending action sequences when they have to rely exclusively on motor cues^23^,^24^. We suggest that these difficulties, which some findings attribute to abnormalities in action chaining^25^,^26^, might actually be closely related to an impaired ability to use kinematic information to detect intention. Evidence for this hypothesis, however, is still lacking^27^. Our quantitative behavioral approach could help reveal such a form of ‘kinematic blindness’.

## Conclusions

Understanding the intentions of others is the basis of social cognition, and is of crucial importance for any species living in groups. Based on the assumption that intentional states are perceptually inaccessible, and thus unobservable^28^, standard theories of social understanding have mainly focused on the contribution of higher level, inferential processes aimed at unveiling ‘‘things that can (not) be seen^29^’’. Our study is the first to quantify the ‘visibility’ of intentions in movement kinematics and demonstrate a measurable relationship between the specific features of observed movements, and the ability to read intention. We anticipate our findingswill yield new insights into the psychology and neurobiology of how we understand other minds, and predict others’ behavior, providing a starting point for more sophisticated models of intention understanding, which incorporate subtle kinematics cues as an information source.

## Materials and Methods

### Motion capture and video recording

Seventeen healthy naive volunteers took part in the action execution study (9 females; mean 28.17 years; range 21–39). All participants were right-handed with normal or corrected-to-normal vision, and with no history of either psychiatric or neurological disorders. The research was approved by local ethical committee (ASL 3 Genovese), and was carried out in accordance with the principles of the revised Helsinki Declaration (World Medical Association General Assembly, 2008). Written informed consent was obtained from each participant.

Participants were tested in a single experimental session lasting approximately 50 minutes. They were seated on a height-adjustable chair, with their right elbows and wrists resting on a table (length = 110 cm; width = 100 cm). In order to guarantee a repeatable start position across participants, they were asked to maintain their forearms in a pronated position, with their right arms oriented in the parasagittal plane passing through the shoulder, and their right hands in a semi-pronated position, with the tips of their thumbs and index fingers on a tape-marked point, placed on the working space. A glass bottle was positioned on the table at a distance of about 46 cm from participants’ midline. Participants’ right hands were outfitted with 20 lightweight retro-reflective hemispheric markers (4 mm in diameter). A near-infrared camera motion capture system with nine cameras (frame rate, 100 Hz; Vicon System) was used to track the hand kinematics. Movements were also filmed from a lateral viewpoint using a digital video camera (Sony Handy Cam 3-D, 25 frames/sec). The video camera was placed at about 120 cm from the starting position of participants’ hands, with the camera view angle directed perpendicularly to body midline. Video camera position and arrangement were kept constant for the duration of the study in order to ensure that one’s hand and the bottle were in full view from the beginning to end of the movement. After data collection, each trial was individually inspected for correct marker identification, and then run through a low-pass Butterworth filter with a 6 Hz cutoff. For data processing and analysis, a custom software (Matlab; MathWorks, Natick, MA) was used to compute two sets of variables.

The first set of variables, expressed with respect to the original frame of reference (i.e., the frame of reference of the motion capture system, termed as global frame of reference; F_global_), included:

a. Wrist Velocity, defined as the module of the velocity of the wrist marker (mm/sec);

b. Wrist Height, defined as the z-component of the wrist marker (mm);

c. Wrist Horizontal Trajectory, defined as the x (i.e. left-right) component of the wrist marker (mm);

d. Grip Aperture, defined as the distance between the marker that was placed on the thumb tip and the marker placed on the tip of the index finger (mm).

To provide a better characterization of the hand joint movements, the second set of variables were expressed with respect to a local frame of reference centered on the hand (i.e., F_local_; see ref. 30, for a detailed description of the F_local_). Within F_local_, we computed the following variables:

e. x-, y-, and z-thumb, defined as x-, y- and z-coordinates for the thumb with respect to F_local_ (mm);

f. x-, y-, and z-index, defined as x-, y- and z-coordinates for the index with respect to F_local_ (mm);

g. x-, y-, and z-finger plane, defined as x-, y- and z-components of the thumb-index plane, i.e., the three-dimensional components of the vector that is orthogonal to the plane. This variable provides information about the abduction/adduction movement of the thumb and index finger, irrespective of the effects of wrist rotation and of finger flexion/extension.

h. x-, y-, and z-dorsum plane, defined as x-, y- and z-components of the radius-phalanx plane. This variable provides information about the abduction, adduction, and rotation of the hand dorsum, irrespective of the effects of wrist rotation.

All variables were calculated only considering the reach-to-grasp phase of the movement, i.e., from the reach onset (i.e. the first time at which the wrist velocity crossed a 20 mm/s threshold) to the grasp offset (i.e. the time at which the wrist velocity dropped below a 20 mm/s threshold). This means that the second part of the movement, starting from the lift of the bottle, was not considered in the kinematic analysis. Each of the 16 variables was expressed with respect to normalized (%) rather than absolute (ms) duration, and was then resampled at intervals of 10% of the normalized movement time.

### Experiment 1: observation of representative movements

Eighteen healthy new volunteers took part in Experiment 1 (9 females; mean 24.78 years; range 20–32). All participants were right-handed with normal or corrected-to-normal vision, and with no history of either psychiatric or neurological disorders. The experiment was carried out in a dimly lit room. Participants sat in front of a 17-inch computer monitor (1280 × 800 resolution, 75 Hz) at a viewing distance of 50 cm. Participants were presented with video clips showing the reach-to-grasp phase of the 100 representative movements resulting from the LDA. Each trial started with a presentation of a screen (1500 ms) informing the participant about the button press for a specific intention. For half of the participants, the Italian word ‘‘beve’’ (to drink) on the left prompted a button press with index finger on the left button of a wireless keyboard touchpad, while the word ‘‘versa’’ (to pour) on the right prompted a button press with middle finger on the touchpad right button. The position of the two words was counterbalanced across participants. This first screen was followed by a green fixation cross (+) at the center of the monitor for 1500 ms. Then, a video-clip showing the reach-to-grasp phase of the action was presented. The duration of the videos varied according to the actual duration of the movement (mean = 1.05 s, se = 0.002 s, range = 0.76 to 1.64 s), which did not differ between intentions (t_98_ = −0.311; p = 0.757). To ensure that movement sequences could be temporally attended (i.e., to provide participants enough time to focus on movement start), 9, 11, or 13 static frames were randomly added at the beginning of each video clip. The task consisted of predicting the intention underlying the observed action by pressing one of the two response buttons. Participants were instructed to respond correctly and as quickly as possible. Participants could respond either during the video, or within a maximum of 3000 ms after the video ended. No feedback was provided to participants at any stage of the experiment. After indicating a response, participants were requested to rate the confidence of their decision on a 4-point scale by pressing a key (from 1 = least confident, to 4 = most confident). They were encouraged to use the entire confidence scale.

The main experiment was performed in four blocks. During each block all of the videos (N = 100) for both intentions were presented. The video presentation was randomized over blocks. In total, 400 trials were shown. At the beginning of the experiment, participants were presented with two sample movements within the action execution experimental setup, so that they could see the phase during which the agent poured the water into the glass, or brought it to his or her own mouth to drink the water. Further, before starting the experiment, participants performed a short practice (8 videos each for the two intentions). In these practice trials, participants first watched the reach-to-grasp phase, and then the entire movement without providing any response. The entire main experiment lasted approximately 50 minutes. Stimuli presentation, timing, and randomization procedures were controlled using E-prime (version 2.0.10.242).

To estimate SDT parameters we labelled by convention grasp-to-drink actions as signal and grasp-to-pour actions as no-signal trials. The combination of hits (i.e., signal responses on signal trials) and false alarms (i.e., signal responses on no-signal trials) with confidence ratings was used to calculate the AUC for each participant. We then submitted AUC values to a one-sample t-test in order to ascertain participants’ ability to distinguish between signal and no signal above the chance/guessing level of 0.5.

#### Classification and regression tree (CART)

The CART model^31^ was developed using SPSS modeler 14.1 software (IBM Corp. USA). CART analysis is a tree-building technique that splits data into increasingly homogenous subgroups by means of a recursive data partitioning. Participants’ responses (to-pour or to-drink) were included in the model as a categorical outcome. All of the kinematics variables described above were used as predictors or ‘independent’ variables. We trained the model on 70% of the data, and tested it on the remaining 30%. Since trees can reach a high level of complexity based on the specific training dataset used to build them, the CART model was pruned, removing bottom-level splits that did not significantly contribute to the tree’s accuracy. We used the SPSS Modeler default standard error rule for pruning.

### Experiment 2: observation of all movements of Subset 1

Eighteen healthy new volunteers took part in Experiment 2 (9 females; mean 24.39 years; range 21–29). Due to accuracy values deviating more than 3 SD from the group average, one participant was excluded from the analysis, resulting in a sample of 17 participants (8 females; mean 24.35 years; range 21–29). As in Experiment 1, all participants were right-handed with normal or corrected-to-normal vision, and with no history of either psychiatric or neurological disorders. All video clips of Subset 1 (grasp-to-pour, N = 128, grasp-to-drink, N = 128) were used in this experiment. As in Experiment 1, each video clip was edited in such a way that only the reach-to-grasp phase of the movement was visible. The experimental procedure was the same as in Experiment 1, with the only difference being that participants were presented with 256 unique video clips (grasp-to-pour, N = 128, grasp-to-drink, N = 128), instead of 100 (grasp-to-pour, N = 50, grasp-to-drink, N = 50). Due to the high number of stimuli, the main experiment was performed in two instead of four blocks. During each block, all of the videos for both intentions were presented, resulting in a total of 512 trials.

The CART model generated in Experiment 1 was used to predict participants’ classification accuracy in Experiment 2. Indeed, a key purpose of classification and regression trees is to allow, based on predictor variables (i.e. kinematic features), accurate predictions of outcome for future experimental samples. There are two kinds of predictions that a classification tree can make: i) a point prediction (i.e., a binary guess about the intention category); and, ii) a distributional prediction (i.e., the probability associated with the point prediction). By combining the two predictions with the actual movement intention, we obtained an overall accuracy prediction for the 256 movements (128 grasp-to-pour and 128 grasp-to-drink) of Subset 1. For those movements for which the intention choice predicted by the model corresponded to the actual intention, we considered the value of the distributional prediction as ‘predicted accuracy’. For those movements for which the intention choice predicted by the model did not correspond to the actual intention, we considered ‘1 - distributional prediction’ as ‘predicted accuracy’. The overall predicted accuracy corresponded to 0.549. Accuracy values of the 17 participants were submitted to a one-sample t-test using 0.549 (i.e. the predicted accuracy) as a test value.

A multiple regression was performed to test the relation between the predictive performance of the model and the participants’ accuracy for each of the 256 videos. We set the mean participants’ accuracy for each video as outcome, and the ‘CART accuracy predictions’ and the ‘subject’ as predictors. ‘Subject’ was treated as a categorical factor using dummy variables. We then removed the between participant variability and obtained a correlation coefficient r_15 _= 0.572; p < 0.001 32.

### Experiment 3: manipulation of intention visibility

Eighteen healthy new volunteers took part in Experiment 3 (10 females; mean 24.50 years; range 22–30). One participant was excluded from the analysis because of accuracy values deviating more than 3 SD from the group average. This resulted in a total of 17 participants (9 females; mean 24.59 years; range 22–30). As in previous experiments, all participants were right-handed with normal or corrected-to-normal vision, and with no history of either psychiatric or neurological disorders.

One hundred video clips of Subset 2 (grasp-to-pour, N = 50, grasp-to-drink, N = 50) were used for Experiment 3. Stimuli selection was driven by the application of the CART model generated in Experiment 1 on this new set of movements. In particular, we selected the 100 video clips that, based on CART prediction, could maximize participants’ performance. The overall predicted accuracy for the selected videos was 0.697. As in Experiments 1 and 2, each video clip was edited so that only the reach-to-grasp phase of the movement would be visible. The experimental procedure was the same as in Experiment 1.

As with Experiment 2, accuracy values of participants were submitted to a one-sample t-test using 0.697 (i.e. the predicted accuracy) as a test value. Finally, we tested whether the movement selection driven by the CART model resulted in a significantly higher participants’ sensitivity. We thus performed an univariate ANOVA, followed by post-hoc tests to compare AUC values of Experiment 3 with those of Experiments 1 and 2.

## Additional Information

**How to cite this article**: Cavallo, A. *et al*. Decoding intentions from movement kinematics. *Sci. Rep.* **6**, 37036; doi: 10.1038/srep37036 (2016).

**Publisher’s note:** Springer Nature remains neutral with regard to jurisdictional claims in published maps and institutional affiliations.

## Supplementary Material

Supplementary Information

Supplementary Video S1

Supplementary Video S2

Supplementary Video S3

Supplementary Video S4

Supplementary Video S5

Supplementary Video S6

Supplementary Video S7
