# Supplementary material for: Decoding intentions from movement kinematics
Source: Sci Rep. 2016 Nov 15;6:37036. doi: 10.1038/srep37036 (PMC5109236; doi:10.1038/srep37036)
Supplement: Supplementary Information [file srep37036-s1.pdf]

Decoding intentions from movement kinematics

Andrea Cavallo[1], Atesh Koul[2], Caterina Ansuini[2], Francesca Capozzi[1] & Cristina Becchio[1,2*]

[1] Department of Psychology, University of Torino, Torino, Italy

[2] C'MON, Cognition, Motion and Neuroscience Unit, Fondazione Istituto Italiano di Tecnologia, Genova, Italy

Supplementary Figure S1. CART model for predicting intention choice from the observation of movements that minimized the within-intention distance. The model was generated using participants' responses as outcome and kinematics features as predictors. The analysis yielded seven decision rules resulting in twenty nodes. Each node provides the total number of trials within the node and the number of 'Grasp-to-pour' and 'Grasp-to-drink' choices. For example, node 3 represents high probability of 'Grasp-to-pour' choice, while node 6 represents high probability of 'Grasp-to-drink' choice.

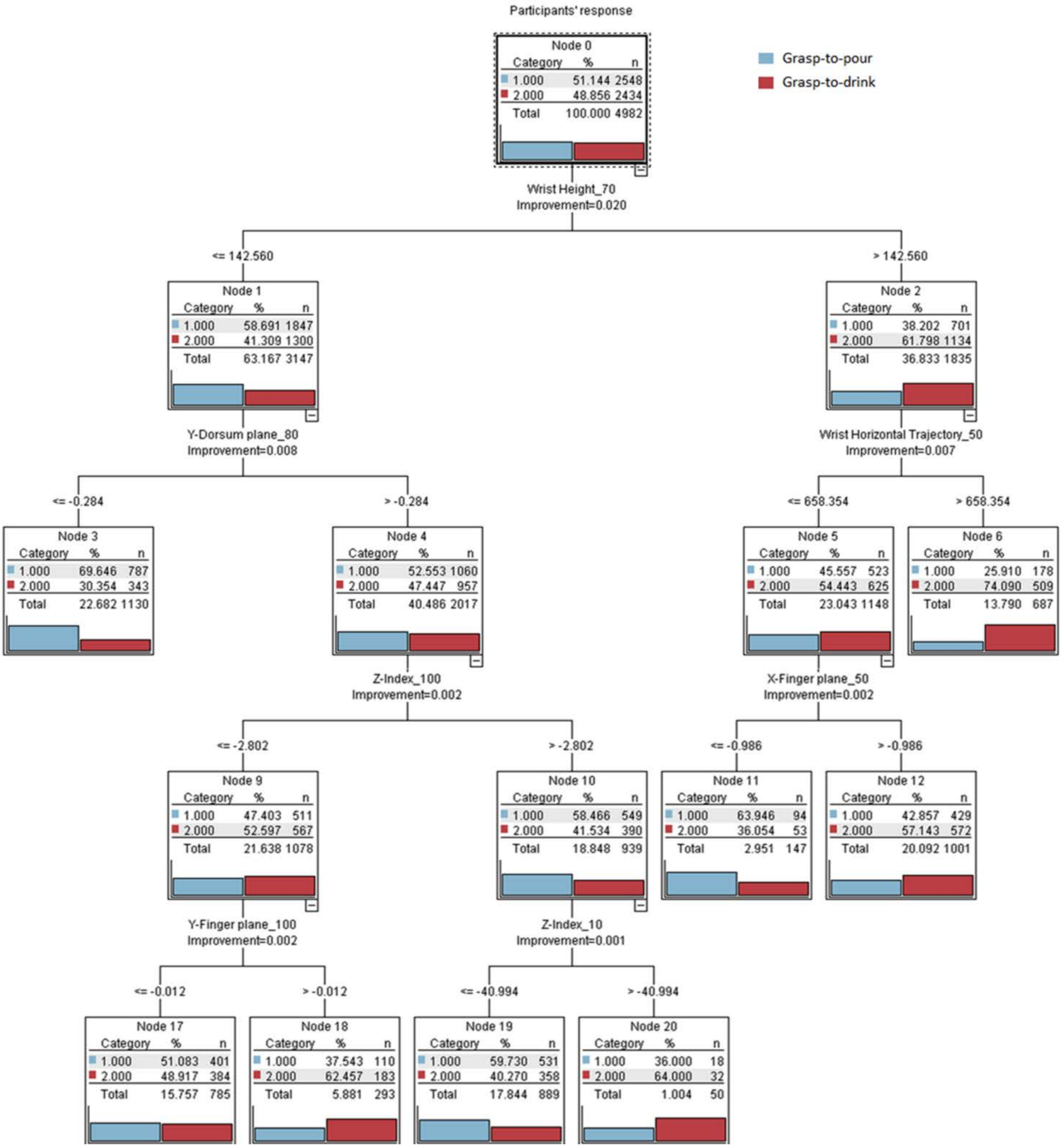

Supplementary Video S1. **Reach-to-grasp phase of a representative movement performed with the intent *to pour* water into a small glass**. Colored dots on the left panel represent the position of 20 lightweight retro-reflective hemispheric markers placed on the participants' right hand and of 4 markers placed on the base of the bottle.

Supplementary Videos S2-S4_Grasp-to-pour. Reach-to-grasp phase of three representative 'to-pour' movements shown in Experiment 1.

Supplementary Videos S5-S7_Grasp-to-drink. Reach-to-grasp phase of three representative 'to-drink' movements shown in Experiment 1.
